# Considerations and anesthetic management of a patient with giant right atrial myxoma: A case report and literature review

**DOI:** 10.1097/MD.0000000000037141

**Published:** 2024-02-16

**Authors:** Yan Qu, Lei Li, Min Deng, Duanyi Song, Min Gao, Guoning Su

**Affiliations:** a Department of Anesthesiology, Affiliated Hospital of Yunnan University, Kunming, Yunnan, PR China; b Department of Radiology, Affiliated Hospital of Yunnan University, Kunming, Yunnan, PR China

**Keywords:** anesthetic management, moderate altitude, right atrial myxoma

## Abstract

**Background:**

Myxoma is a common type of primary cardiac tumor. However, there are few researches to illustrate challenge of safely inducing anesthesia in a patient with a giant right atrial myxoma at moderate altitude.

**Patient concerns and diagnoses:**

A 54-year-old female patient lived in a city with an average altitude of 1932 m with scheduled surgical treatment for giant right atrial myxoma, prompting discussions on appropriate anesthesia modalities given her prolonged residence at moderate altitude.

**Methods and Results:**

Considering the potential impact of moderate altitude on perioperative management, this study emphasizes the necessity of adequate volume preload therapy and the utility of transthoracic echocardiography or transesophageal echocardiography to prevent hemodynamic compromise. Furthermore, it highlights the unique consideration that, post-tumor removal, hypotension may not necessarily lead to decreased oxygen saturation in these patients.

**Conclusion:**

This case underscores the importance of avoiding hypotension, as pre-tumor resection blood pressure maintenance primarily determines blood oxygen concentration. Additionally, it sheds light on the intriguing observation that post-tumor removal hypotension may not result in decreased oxygen saturation. These findings have significant implications for the perioperative care of patients with giant right atrial myxoma at moderate altitudes.

## 1. Introduction

Since initially reported a case of 50-year-old patient with right atrial myxoma by Coates and Drake^[1]^ in 1958, there were about 150 cases of myxoma of the heart been described in the world literature. Given these clinical pictures, the anesthesiologist must pay attention to hypoxemia and severe hypotension during induction of anesthesia and operation, as Moritz and Azad^[2]^ illustrated. Whereas even sporadic myxomas can pose many anesthetic and surgical challenges, familial case often is associated with additional problems that arise from moderate altitude. The current literature contains few recommendations to guide anesthetic management in such case.

“Moderate altitude” means anything from 1500 to 2500 m (4900–8000 ft) according to the American Heart Association. The patient in this case had long lived in Kunming, a city with an average altitude of 1932 m above sea level where the surgery performed. With prolonged exposure to altitude, the peripheral chemoreceptors become more sensitive to hypoxia, thus stimulating a heightened sympathetic activity and an increased ventilation, despite the progressive increase in arterial blood O_2_ content. These unique characteristics make special attentions to anesthesia-induced hypoxemia in this patient.

The following case illustrates the challenge of safely inducing anesthesia in a patient with a giant right atrial myxoma, considering potential compensatory capacity for hypoxia and characteristics of intraoperative hemodynamic changes at moderate altitude.

## 2. Case presentation

A 54-year-old female patient was admitted to hospital with recurrent chest tightness and pain without an obvious cause for more than 2 years. The pain was not intense and could be relieved itself after a few seconds. She had no difficulty breathing or sleeping. The patient initially presented to a hospital in March 2021 with symptoms of recurrent angina and was diagnosed with myocardial ischemia. She was given oral medication to treat myocardial ischemia, but the symptoms still recurred. However, subsequent clinical and diagnostic investigations failed to reveal the underlying right atrial myxoma (specific details regarding diagnostic tests performed during initial workup unknown). In September 2022, her symptoms worsened and she returned to that hospital again for treatment. Coronary angiography revealed 20% to 30% stenosis in the proximal coronal segment of right coronary artery. Transthoracic echocardiography (TTE) examination of the heart indicated an echoic mass like parenchyma in the right atrium. Given the size of the right atrial myxoma, she was suggested to be transferred to our hospital for further treatment.

**Figure 1.**
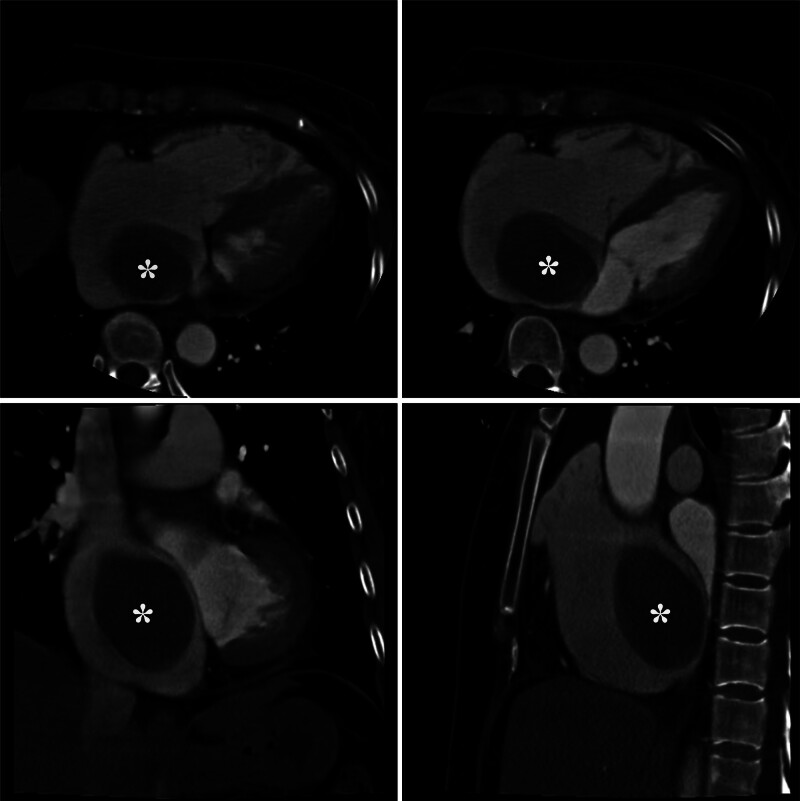
The contrast computerized tomographic scan revealed an abnormal shadow defect (asterisks).

**Figure 2.**
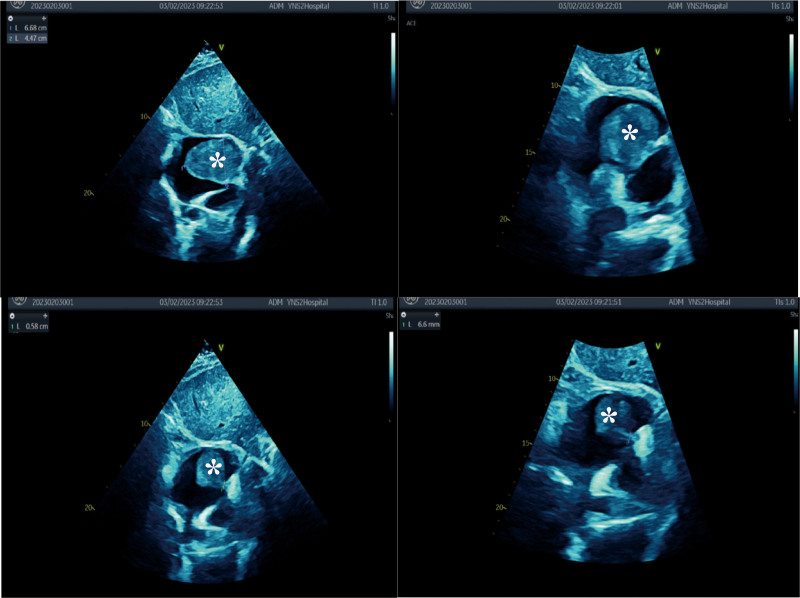
Three-dimensional transthoracic echocardiography showed a 66.8 mm × 44.7 mm voluminous mass (asterisks) in the right atrium.

The computed tomography scan showed a 59°×°41mm large mass occupying most of the right atrial cavity (Fig. 1). Surveillance TTE revealed a large mobile mass consistent with myxomas in the right atrium and tricuspid insufficiency (Fig. 2). It was approximately 68 × 44.7 mm with a tip attached to the coronary sinus opening and originated from the fossa ovalis as seen on subsequent transesophageal echocardiography (TEE). The right atrium and ventricle were dilated. Laboratory reports included a hemoglobin of 115 g/L, hematocrit of 36.6%, and arterial blood gas (ABG) was values breathing air (with 16% O_2_) of pH 7.4, PaO_2_ 77.8 mm Hg, PaCO_2_ 38.4 mm Hg, and O_2_ saturation of 89%.

The patient was brought to the operating room, administered 100% O_2_ via face mask while monitors were applied. Pulse oximetry revealed an oxygen saturation (SpO_2_) of 93%. Noninvasive blood pressure (BP) was 110/73 mm Hg, while the heart rate 65 beats/min. Before induction, a central venous catheter was inserted from the right internal jugular vein under ultrasound guided and invasive BP measurement was performed. The average central venous pressure was 12 cm H_2_O. To ensure maintenance of circulation and to induce anesthesia safely, 500 mL of crystalloid were administered to maintain preload, and inotropic support was avoided to prevent obstruction of the tricuspid valve.

**Figure 3.**
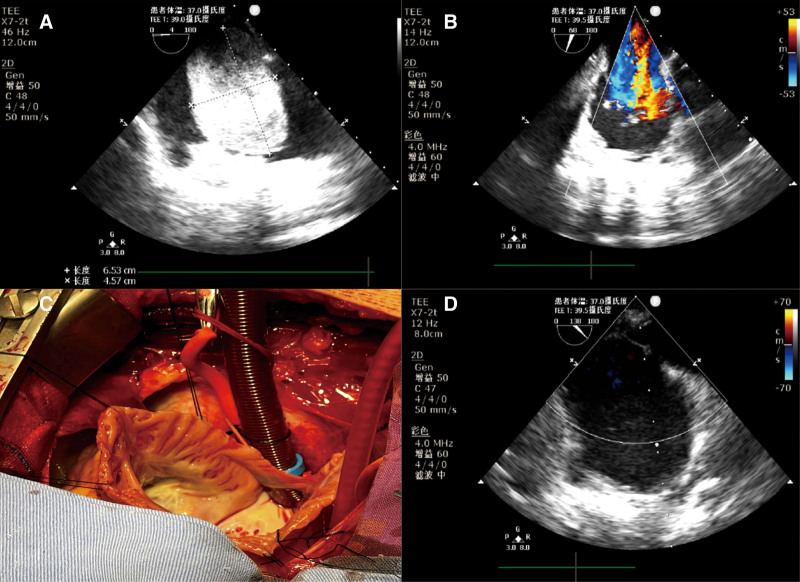
(A) Transthoracic echocardiography reveals a right atrial mass with inhomogeneous echo (65.3 mm × 45.7 mm). (B) Apical 4-chamber view with color flow mapping, preoperatively, showing tricuspid regurgitation. (C) Intraoperative photograph showing the myxoma been resected in the right atrium. (D) Apical 4-chamber view with color flow mapping, postoperatively, showing none tricuspid regurgitation.

Anesthesia was induced with 5 mg of midazolam, 50 mcg of sufentanil, 10 mg of etomidate, and 8 mg of vecuronium. Before intubation, BP dropped to 85/50 mm Hg and SpO_2_ fell to 75% even 500 mL of crystalloid were administered. Phenylephrine 50 mcg was given while ABG analysis suggested pH 7.463, PaO_2_ 79.6 mm Hg, PaCO_2_ 38.4 mm Hg, and O_2_ saturation of 88% via mask pressurized 100% oxygen delivery. TEE showed the mass caused moderate tricuspid valve regurgitation (Fig. 3A, 3B and Videos 1 and 2, Supplemental Digital Content, http://links.lww.com/MD/L636; http://links.lww.com/MD/L638). Pulmonary vascular resistance can be kept low using lung-protective ventilatory strategies, optimum positive end-expiratory pressure, and 60% oxygen.

After a median sternotomy, the superior and inferior vena cava as well as the aortic cannulations were performed for cardiopulmonary bypass. A huge dark-red to yellow color, vascularized, gelatinous mass filling the right atrium was found. It was attached to the coronary sinus opening below the fossa ovalis by a thin stalk. Cardiac Surgeons delivered and released the mass, identified and resected the stalk, and then cauterized the base of the stalk. During resection of the tumor, atrial septal defect is created which at times necessitates patch closure. Hence, the aorta should be cross clamped during the surgery. De Vega surgery was performed as well (Fig. 3C) and the tricuspid insufficiency was disappeared after surgery (Fig. 3D). Specifically, when the mean arterial pressure was maintained above 60 mm Hg, the PaO_2_ was up to 150 mm Hg before the tumor was removed. After removal of the tumor, PaO_2_ exceeded 200 mm Hg even if BP was below 90/50 mm Hg.

**Figure 4.**
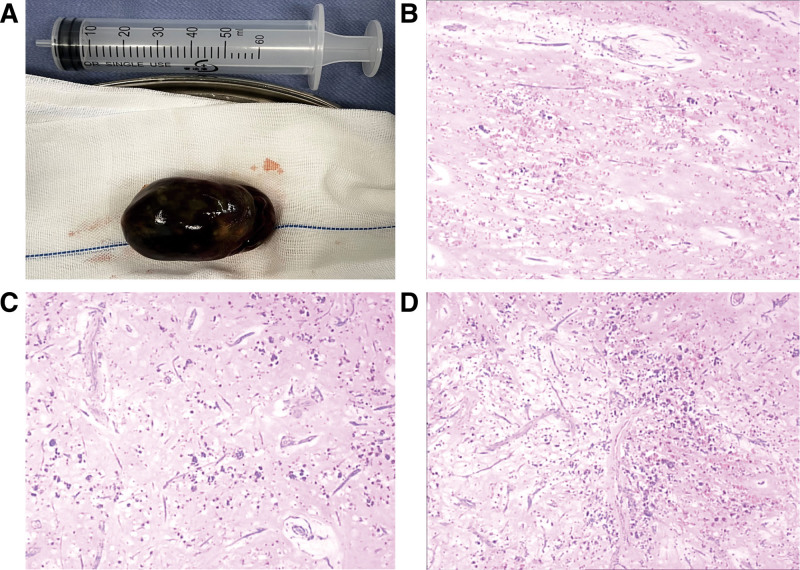
(A) Gross examination of the surgical specimen reveals a red-colored mass (75 × 45 × 45 mm) with multiple-grey spots, an irregular and hard surface. (B, C and D) The histopathologic examination confirms the diagnosis of calcified myxoma.

The patient was weaned from cardiopulmonary bypass successfully after 140 minutes and remained hemodynamically stable without inotropic or vasopressor support. She was extubated 4 hours later. Histopathology confirmed the diagnosis of right atrial myxoma. The mass measured 75 × 45 × 45 mm. Microscopy showed stellate cells in myxoid stroma with areas of hemorrhage and necrosis. No malignancy was detected (Fig. 4).

## 3. Discussion

Myxoma is a common type of primary benign cardiac tumor. Current data shows that approximately 75% of them is located in the left atrium, 23% in the right atrium, and only 2% in the ventricles. The clinical features of myxomas are determined by their location, size, mobility and fragility. Patients with tumors on the right side of the heart may present with shortness of breath and easily fatigue, as in our case. Back and forth movement of a tumor between the atrium and ventricle can also impede valve closure or damage the atrioventricular valve apparatus with tendon rupture and lead to mitral or tricuspid regurgitation in addition to stenosis.

Right atrial myxomas may mimic constrictive pericarditis by producing functional stenosis of the tricuspid valve, with increased right atrial pressure. Ventricular myxomas may mimic the aortic or pulmonic valve stenosis because of the narrowing of the left or right ventricular outflow tract, respectively, and may also cause syncope. In collected series and case reports of myxoma, the most common symptom of right atrial myxoma is dyspnea. Dyspnea might also be caused by increased pulmonary dead space (West Zone I) from reduced pulmonary blood flow. Hypoxemia, indicated by low arterial SpO_2_ ranged from 46% to 97%.^[3,4]^ This might be due to several reasons, including low cardiac output caused by tricuspid valve obstruction, right-to-left cardiac shunt through a patent foramen oval, or pulmonary embolism from tumor fragmentation.

As Melnyk et al^[5]^ found, myxomas in the right atrium may pose a risk of embolization during central venous or pulmonary artery catheterization. Embolization of right atrial myxoma can lead to pulmonary embolism. Constitutional symptoms may include fever, weight loss, and anemia mimicking infective bacterial endocarditis or systemic vasculitis.^[6]^ Although 2-dimensional TTE has its limitations, a central venous catheter (internal jugular vein) was inserted under visualization with TTE to prevent embolization of tumor fragments that originate from contact between a catheter and the tumor into the pulmonary vessels.^[7]^ In this patient, tumor embolization seems unlikely in light of a normal TTE or TEE scan and ultrasound-guided internal jugular vein placement. No tumor fractures or embolization was observed during the operation and consequently the patient’s hemodynamics were stable.

Right-sided myxomas may cause pulmonary emboli and severe congestive heart failure by obstructing blood flow across the tricuspid valve depending on their size and mobility.^[8,9]^ Preventing these severe phenomena is essential for the successful anesthetic management during excision of the tumor. From this case report, adequate volume preload therapy, anesthetic implications and usefulness of the TTE or TEE to avoid hemodynamic compromise and lethal events have been shown for the intraoperative management of patients undergoing excision of a giant cardiac tumor in the moderate altitude environment.

Prolonged altitude acclimatization leads to physiological adaptations that improve oxygen delivery during normal activity at moderate high altitude, but also amplify the body’s response to acute hypoxemia as can occur during anesthesia and surgery. Specifically, peripheral chemoreceptors become hypersensitive to changes in oxygen levels, increasing sympathetic activity and ventilation. However, arterial oxygen content also increases over time at altitude, as does hemoglobin concentration and oxygen carrying capacity. Furthermore, this case demanded meticulous management of oxygenation, ventilation, hemodynamics and acid-base status to avoid instability in a patient whose compensatory mechanisms for hypoxemia have been set at a “higher gear” by long term moderate altitude exposure. Moreover, although some degree of pulmonary hypertension is common at altitude, fluid overload or hypertension risks right heart failure or end organ damage in these patients.

This patient developed hypotension during induction of anesthesia, which can be caused by low cardiac output due to obstruction of right ventricular filling in addition to peripheral vasodilation caused by anesthetic drugs. There was no evidence of right-to-left shunt preoperatively or intraoperatively. In addition, maneuvers such as intermittent positive pressure ventilation help maintain lung volumes and oxygenation, especially when diaphragmatic function is impaired under anesthesia or during open chest conditions.

The limitations of the study are listed below. The study relied on collected serial data and case reports, which may have inherent biases and limitations in terms of data quality and generalizability. On the other hand, we mentioned that the patients lived at moderate altitude for a long period of time, which may introduce confounding variables related to physiological adaptation, making it challenging to isolate the specific effects of cardiac tumors.

In conclusion, given this patient’s long-term residence in moderate altitude for a long time, the effects of moderate altitude must be considered in her perioperative management. We discussed specific clinical features associated with moderate altitude environment, highlighting the importance of adequate volume preload therapy, anesthetic implications and usefulness of the TTE or TEE to avoid hemodynamic compromise. Lastly, it delved into the challenges of anesthetic management during excision of the tumor in a moderate altitude environment, emphasizing the need for meticulous perioperative care and highlighting the impact of physiological adaptations on patient management.

## Author contributions

Data curation: Duanyi Song.

Resources: Min Deng, Min Gao.

Supervision: Guoning Su.

Visualization: Yan Qu.

Writing – original draft: Yan Qu, Lei Li.

Writing – review & editing: Yan Qu.

## Supplementary Material





## References

[1] Coates EO Jr. Drake EH. Myxoma of the right atrium, with variable right-to-left shunt; clinical and physiologic observations and report of a case with successful operative removal. N Engl J Med. 1958;259:165–9. 13566441 10.1056/NEJM195807242590404

[2] Moritz HA Azad SS. Right atrial myxoma: case report and anaesthetic considerations. Can J Anaesth. 1989;36:212–4. 2706714 10.1007/BF03011447

[3] Nasser WK Davis RH Dillon JC. Atrial myxoma. I. Clinical and pathologic features in nine cases. Am Heart J. 1972;83:694–704. 5025595 10.1016/0002-8703(72)90411-5

[4] Morrissey JF Campeti FL Mahoney EB. Right atrial myxoma. report of two cases and review of the literature. Am Heart J. 1963;66:4–14. 14045987 10.1016/0002-8703(63)90063-2

[5] Melnyk V Hackett PJ Subramaniam K. Complex considerations and anesthetic management in patient with multiple intracardiac myxomas. J Cardiothorac Vasc Anesth. 2018;32:1374–6. 28017676 10.1053/j.jvca.2016.09.036

[6] Saadeh AM Hijazi EM Saadeh NA. Right atrioventricular myxoma presenting with recurrent syncopal attacks. Am J Case Rep. 2021;22:e927874. 33561115 10.12659/AJCR.927874 PMC7883939

[7] Tagawa T Okuda M Sakuraba S. Anesthetic management of a patient with giant right atrial myxoma. J Cardiothorac Vasc Anesth. 2010;24:532–3. 10.1053/j.jvca.2009.05.021 19640741

[8] Reynen K. Cardiac myxomas. N Engl J Med. 1995;333:1610–7. 7477198 10.1056/NEJM199512143332407

[9] Parsons AM Detterbeck FC. Multifocal right atrial myxoma and pulmonary embolism. Ann Thorac Surg. 2003;75:1323–4. 12683591 10.1016/s0003-4975(02)04563-0

